# Loneliness and Cognitive Functioning Over Time: Using Ambulatory Cognitive Assessment

**DOI:** 10.1093/geroni/igaa057.1869

**Published:** 2020-12-16

**Authors:** Jee-eun Kang, Karra Harrington, Martin Sliwinski

**Affiliations:** 1 Pennsylvania State University, University Park, Pennsylvania, United States; 2 Penn State University, University Park, Pennsylvania, United States

## Abstract

Loneliness has been investigated as a risk factor for cognitive health, but results were inconsistent. This study used three measurement bursts of ambulatory cognitive assessment to determine whether loneliness affects longitudinal changes in cognitive functioning in daily life. At each burst, participants performed cognitive assessment five times a day for 14 days. 138 adults (Mage=49.4) who completed all three bursts were included in this study.

Growth curve modeling showed that, on average, scores of cognitive functioning were improved across a 2 year period (p<.001). The chronic lonely group (in the highest tertile at all 3 bursts) showed less improvement in scores compared to non-lonely people (p<.01), although there was no difference in cognitive functioning at the baseline between two groups. This study indicates that we need a repeated measurement of cognitive functioning and longitudinal approach to detect the effect of chronic loneliness on the rate of cognitive change. Part of a symposium sponsored by the Measurement, Statistics, and Research Design Interest Group.

